# Brazilian Green Propolis Extract Synergizes with Protoporphyrin IX-mediated Photodynamic Therapy via Enhancement of Intracellular Accumulation of Protoporphyrin IX and Attenuation of NF-κB and COX-2

**DOI:** 10.3390/molecules22050732

**Published:** 2017-05-04

**Authors:** Cheng-Cheng Wang, Yu-Xuan Wang, Nian-Qin Yu, Die Hu, Xiao-Yan Wang, Xing-Guang Chen, You-Wei Liao, Jing Yao, Hao Wang, Ling He, Liang Wu

**Affiliations:** 1 School of Pharmacy, China Pharmaceutical University, Nanjing 210009, China; w940984614@hotmail.com; 2 School of Life Science and Technology, China Pharmaceutical University, Nanjing 210009, China; w597507666@hotmail.com; 3 Jiangsu Key Laboratory of Drug Screening, China Pharmaceutical University, Nanjing 210009, China; shizi14tian@163.com (N.-Q.Y.); hudiesunny@outlook.com (D.H.); wxy94@hotmail.com (X.-Y.W.); starlight_c@outlook.com (X.-G.C.); lyw0206031@hotmail.com (Y.-W.L.); yaoyaossnh@163.com (J.Y.); wanghao@cpu.edu.cn (H.W.); 4 State Key Laboratory of Natural Medicines, China Pharmaceutical University, Nanjing 210009, China; 5 Department of Natural Medicinal Chemistry, China Pharmaceutical University, Nanjing 210009, China; 6 Department of Pharmacology, China Pharmaceutical University, Nanjing 210009, China

**Keywords:** Brazilian green propolis, synergy, photodynamic therapy, cancer, inflammation, apoptosis

## Abstract

Brazilian green propolis (BGP) is noted for its impressive antitumor effects and has been used as a folk medicine in various cultures for many years. It has been demonstrated that BGP could enhance the cytotoxic effect of cytostatic drugs on tumor cells. Photodynamic therapy (PDT) is a therapeutic approach used against malignant cells. To assess the synergistic effect of BGP extract on protoporphyrin IX (PpIX)-mediated photocytotoxicity, MTT assays were performed using A431 and HeLa cells. TUNEL assay and Annexin V-FITC/PI staining were performed to confirm the induction of apoptosis. Western blotting analysis was performed to examine the pro-apoptotic proteins, anti-apoptotic proteins and inflammation related proteins in A431 cells. Intracellular accumulation of PpIX was examined by flow cytometry. The synergistic effect of BGP extract in PpIX-PDT was also evaluated with a xenograft model. Our findings reveal that BGP extract increased PpIX-mediated photocytotoxicity in A431 and HeLa cells. PpIX-PDT with BGP extract treatment resulted in a decrease in Bcl-xL and an increase in NOXA, Bax and caspase-3 cleavage. The protein expression levels of p-IKKα/β, NF-κB and COX-2 were upregulated by PpIX-PDT but significantly attenuated when in combination with BGP extract. BGP extract was also found to significantly enhance the intracellular accumulation of PpIX in A431 cells. BGP extract increased PpIX-mediated photocytotoxicity in a xenograft model as well. Our findings provide evidence for a synergistic effect of BGP extract in PpIX-PDT both in vitro and in vivo.

## 1. Introduction

As a natural product, propolis has been known and used as a traditional folk medicine in various cultures for thousands of years. It is composed mainly of wax and resins. The latter are derived from plants and contain substances with biological activity. The composition of propolis is variable, and many plant substances have been identified for the first time in propolis [1].

Due to the rich and diverse flora in Brazil, propolis samples collected from this country show distinct colors and chemical compositions depending on the local flora at the site of collection [2]. Brazilian Green Propolis (BGP) is collected from the Minas Gerais region of Brazil. This region is home to a unique plant species, *Baccharis dracunculifolia* D.C., which produces the best grade of propolis with the highest level of flavonoids and artepillin C. The Africanized honeybee from this region can produce propolis from the unexpanded leaf buds of *Baccharis dracunculifolia* D.C. plant. Only this bee species has the capacity to produce BGP with concentrated artepillin C, a powerful constituent not found in propolis from other regions.

BGP has been reported to have a wide range of biological properties including anti-bacterial [3], anti-inflammatory [4,5,6,7], anti-hypertensive [8,9], anti-hyperlipidemic [10], antioxidant [11] and antitumor [12,13,14,15,16,17] activities. BGP was shown to suppress the hypoxia-induced inflammatory responses through inhibition of the nuclear factor-kappa B (NF-κB) activation in microglia [7]. Artepillin C, a specific bioactive component of BGP, was shown to decrease the activity of NF-κB and potentiate the tumor necrosis factor (TNF)-related apoptosis on LNCaP prostate cancer cells [17]. Additionally, an ethanolic extract of BGP was reported to sensitize prostate cancer cells to tumor necrosis factor-related apoptosis-inducing ligand (TRAIL)-induced apoptosis [16].

Photodynamic therapy (PDT) is a clinically approved therapeutic approach that can exert selective cytotoxic activity against malignant cells. Classical PDT involves the administration of two individually non-toxic components: a photosensitizing agent followed by illumination with a laser which has a wavelength specific to the absorbance band of the photosensitizer. Most of the photosensitizers used in cancer therapy are tetrapyrroles, similar to that of the protoporphyrin contained in hemoglobin [18]. As a classic photosensitizer, protoporphyrin IX has been widely used in PDT.

It has been established that PDT could induce inflammation. Elevated levels of inflammation-related molecules within PDT-targeted tissue, such as NF-κB and cyclooxygenase-2 (COX-2), could reduce the antitumor effectiveness of PDT by facilitating survival of residual tumor cells, [18,19,20,21,22]. NF-κB is a major transcription factor that regulates various cell processes, such as apoptosis, inflammation, proliferation, angiogenesis and immunity [23]. COX-2 is overexpressed in many types of cancer and is considered to be associated with reduced patient survival [24]. It has been reported that increased NF-κB and COX-2 expression are some of the molecular factors that contribute to tumor recurrence [18,25,26]. Therefore, inhibiting NF-κB or COX-2 activation might be strategies to improve the tumoricidal efficiency of PDT. It has been demonstrated that dihydroartemisinin enhanced PDT-induced growth inhibition and apoptosis via deactivating PDT-induced NF-κB activation [27]. Furthermore, blockage of COX-2 expression has been shown to facilitate PDT-induced apoptosis [28].

Given its impressive antitumor and anti-inflammatory properties, our study investigates the synergistic effect of BGP extract in PpIX-mediated PDT (PpIX-PDT) both in vitro and in vivo, and explores its potential mechanisms.

## 2. Results

### 2.1. Phytochemical Analysis of Extracts of BGP by HPLC-UV

**Figure 1:**
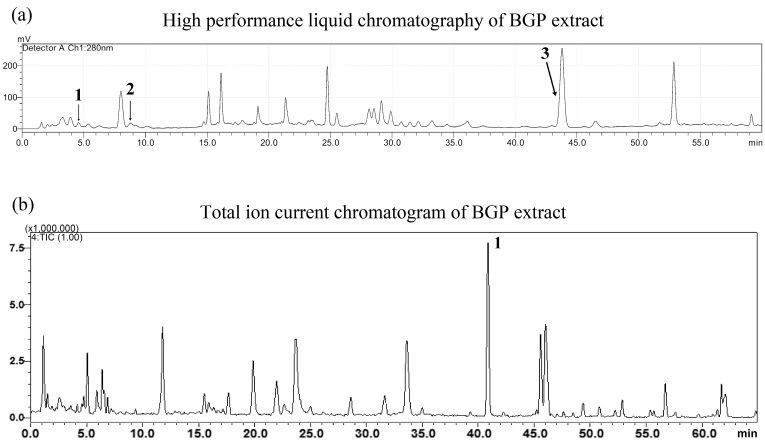
(**a**) HPLC chromatogram of Brazilian green propolis (BGP) extract. (**1**: *p*-coumaric acid, Rt: 4.86 min; **2**: quercetin, Rt: 8.65 min; **3**: artepillin C, Rt: 44.0 min) (**b**) LC-IT-TOF-MS total ion current chromatogram (TIC) of BGP extract in the negative ion mode. **1**: artepillin C, Rt: 42.0 min (PubChem CID 5472440).

The chemical composition of the BGP extract was analyzed by HPLC-UV and a total ion current chromatogram. The content of *p*-coumaric acid (1), quercetin (2) and artepillin C (3) in BGP extracts was about 2.26 mg/g, 2.17 mg/g and 8.23 mg/g, respectively (Figure 1).

### 2.2. BGP Extract Increased PpIX-mediated Photocytotoxicity through Induction of Apoptosis

**Figure 2:**
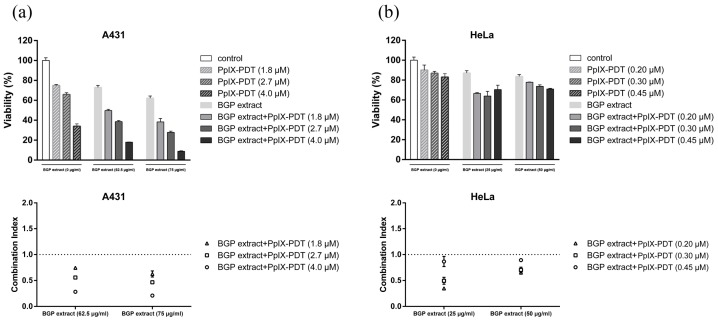
Brazilian green propolis (BGP) extract enhanced protoporphyrin IX (PpIX)-mediated photocytotoxicity in vitro. Combined effects of BGP extract and PpIX-mediated PDT (PpIX-PDT) in A431 and HeLa cells were tested by MTT according to the methods described above. (**a**) A431 cells were incubated with several PpIX concentrations (1.8, 2.7 and 4.0 μM) employing the BGP extract (62.5 and 75 μg/mL); (**b**) For HeLa cells, the concentrations of PpIX were 0.20, 0.30 and 0.45 μM employing the BGP extract (25 and 50 μg/mL). All groups were illuminated by laser. Results were expressed as viability relative to the control. The combination index (CI) corresponded with the data of viability. CI is a quantitative measure of the degree of drug interaction in terms of additive effect (CI = 1), synergism (CI < 1), or antagonism (CI > 1). Data were expressed as mean ± SEM of three independent experiments.

To assess the synergistic effect of BGP extract on PpIX-mediated photocytotoxicity, MTT assays were performed using A431 and HeLa cells. In the presence of different BGP extract dosages (62.5 and 75 μg/mL), the cell toxicity of A431 cells after PpIX-PDT exposure (PpIX = 1.8, 2.7 and 4.0 μM) is shown in Figure 2a.

48 h after illumination, a significant increase of cell toxicity could be observed in the combination group, compared with the BGP extract or PpIX-PDT alone group (Figure 2a). Similar results were observed on HeLa cells in Figure 2b (BGP extract = 25 and 50 μg/mL, PpIX = 0.20, 0.30 and 0.45 μM). The CI values indicated a synergistic enhancement of cytotoxicity in both cells (Figure 2). As 75 μg/mL BGP extract resulted in a significant enhancement of PpIX-PDT-induced cytotoxicity in A431 cells, we adopted 75 μg/mL BGP extract combined with PpIX-PDT in the subsequent analyses.

**Figure 3:**
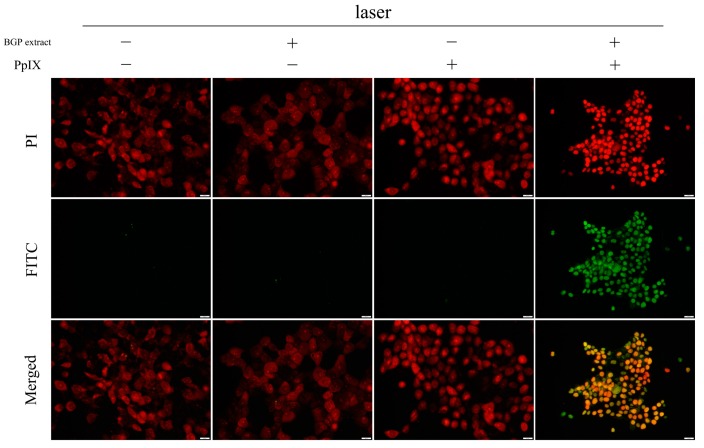
Apoptotic detection by TUNEL assay. PI (red) stained the nucleus while FITC (green) labeled the apoptotic cells. Images were captured by a confocal scanning laser microscope. Scale bar: 50 μm.

**Figure 4:**
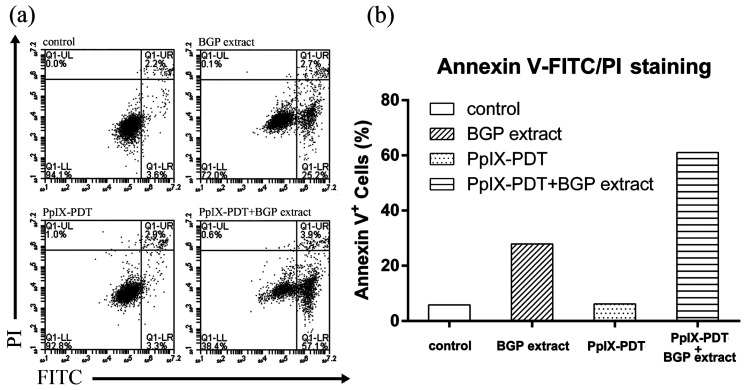
Annexin V-FITC/PI staining of A431 cells. (**a**) Cells were treated with or without Brazilian green propolis (BGP) extract in protoporphyrin IX-mediated photodynamic therapy (PpIX-PDT) and stained using Annexin V-FITC/PI staining assay before detection by a BD Accuri^TM^ C6 flow cytometer. All groups were illuminated by laser; (**b**) The percentages of apoptotic cells.

A TUNEL assay was performed to further confirm the induction of apoptosis by BGP extract on PpIX-mediated photocytotoxicity. As shown in Figure 3, only the combination group exhibited FITC positive signal (green) while the individual treatments were negative, suggesting an enhanced induction of apoptosis by BGP extract in PpIX-PDT. The synergistic effect was further demonstrated by Annexin V-FITC/PI staining (Figure 4), confirming the induction of apoptosis.

**Figure 5:**
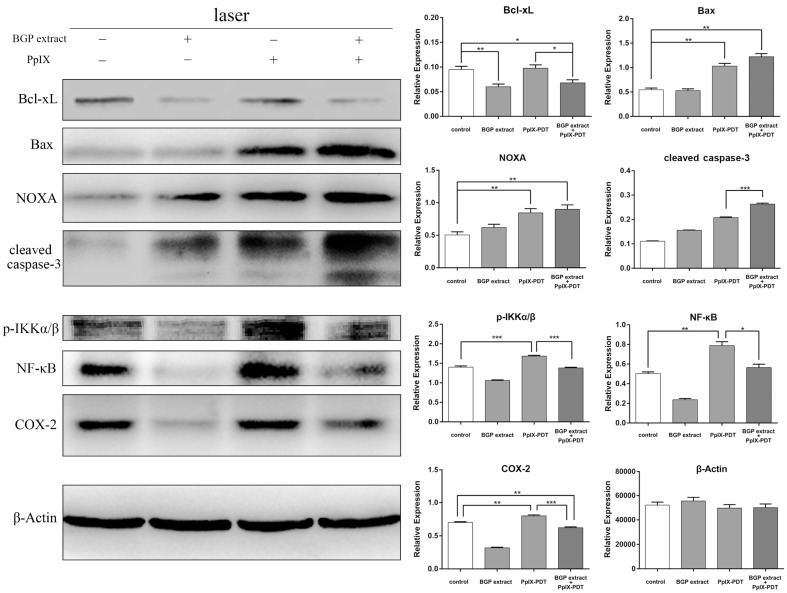
Apoptosis and NF-κB-related proteins expression. Western blotting analysis of the expression of Bcl-xL, Bax, NOXA, cleaved-caspase-3, p-IKKα/β, NF-κB and COX-2, from cell homogenates, with β-Actin as a protein loading control. All groups were illuminated by laser. Data represent mean ± SEM (*n* = 3). * *p* < 0.05, ** *p* < 0.01, *** *p* < 0.001.

To investigate the mechanism of BGP extract induced synergistic enhancement of apoptosis in PpIX-PDT, western blotting analysis was performed to examine the pro-apoptotic proteins (Bax, NOXA and cleaved-caspase-3) and anti-apoptotic protein (Bcl-xL) in A431 cells. When cells were treated with PpIX-PDT, upregulation of Bax and NOXA were induced, while the level of Bcl-xL was unchanged (Figure 5). PpIX-PDT with BGP extract treatment resulted in a decrease in Bcl-xL and an increase in NOXA and Bax in comparison with PpIX-PDT alone treatment (Figure 5). Furthermore, cleaved caspase-3 significantly increased in the combination group compared to the controls, indicating the enhanced activation of apoptotic pathway (Figure 5).

### 2.3. BGP Extract Attenuated PDT-Mediated Elevation of NF-κB and COX-2

PDT could induce NF-κB activation, thereby playing a negative role in the induction of apoptosis. Knowing that propolis could inhibit NF-κB activity, we set out to investigate if BGP extract could enhance the anti-tumor effect of PpIX-PDT on A431 cells via inhibition of NF-κB. Western blotting analysis was performed to examine NF-κB related protein and its downstream proteins expression in A431 cells. The protein expression levels of p-IKKα/β, NF-κB and COX-2 were upregulated by PpIX-PDT but significantly attenuated when in combination with BGP extract (Figure 5) indicating that BGP extract reduced PpIX-PDT induced inflammation.

### 2.4. BGP Extract Enhanced the Anti-tumorigenicity of PpIX-PDT in a Xenograft Model

**Figure 6:**
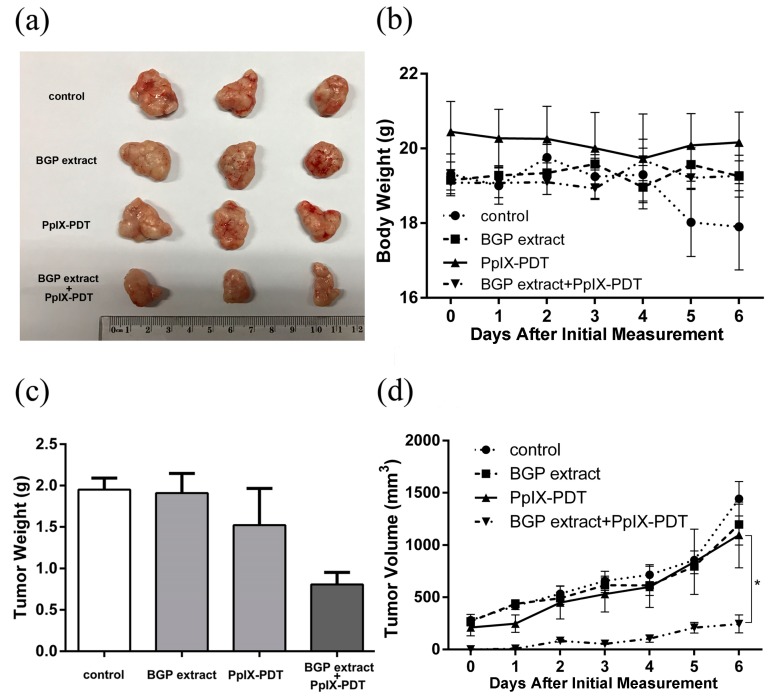
Brazilian green propolis (BGP) extract enhanced the anti-tumorigenicity of protoporphyrin IX-mediated photodynamic therapy (PpIX-PDT) in a BALB/c-nu mice xenograft model. A total of 3 × 10^6^ A431 cells, pretreated with BGP extract (75 μg/mL) and PpIX-PDT (2.7 μM), either alone or in combination, were implanted subcutaneously into BALB/c-nu mice (6 per group). All groups were illuminated by laser. (**a**) The representative images of tumors after two-week development; (**b**,**d**) Body weight and tumor volume (* *p* < 0.05) of mice were measured once per day after one-week development; (**c**) Mice were sacrificed 2 weeks after implantation and tumor weight was measured.

To further assess the synergistic effect of BGP extract and PpIX-PDT, in vivo tumorigenicity after PDT pretreatment was assayed. As shown in Figure 6, six days after inoculation of the pretreated A431 cells, the body weight of the control group decreased while in other groups it kept steady. As expected, the tumor volume was lowest with BGP extract-treated A431 cells following PpIX-PDT; while BGP extract treatment or PpIX-PDT alone did not affect tumor growth in BALB/c-nu mice (Figure 6). Subsequently, the tumor burdens were measured. The results showed that the tumors in BGP extract-combined PpIX-PDT group were significantly distributed in lower tumor volume ranges compared with BGP extract group, PpIX-PDT alone group or the control group (Figure 6). The tumor weight was also decreased in the BGP extract-combined PpIX-PDT group (Figure 6). These results were consistent with the findings of the in vitro assays.

### 2.5. BGP Extract Increased Intracellular Accumulation of PpIX in PDT

**Figure 7:**
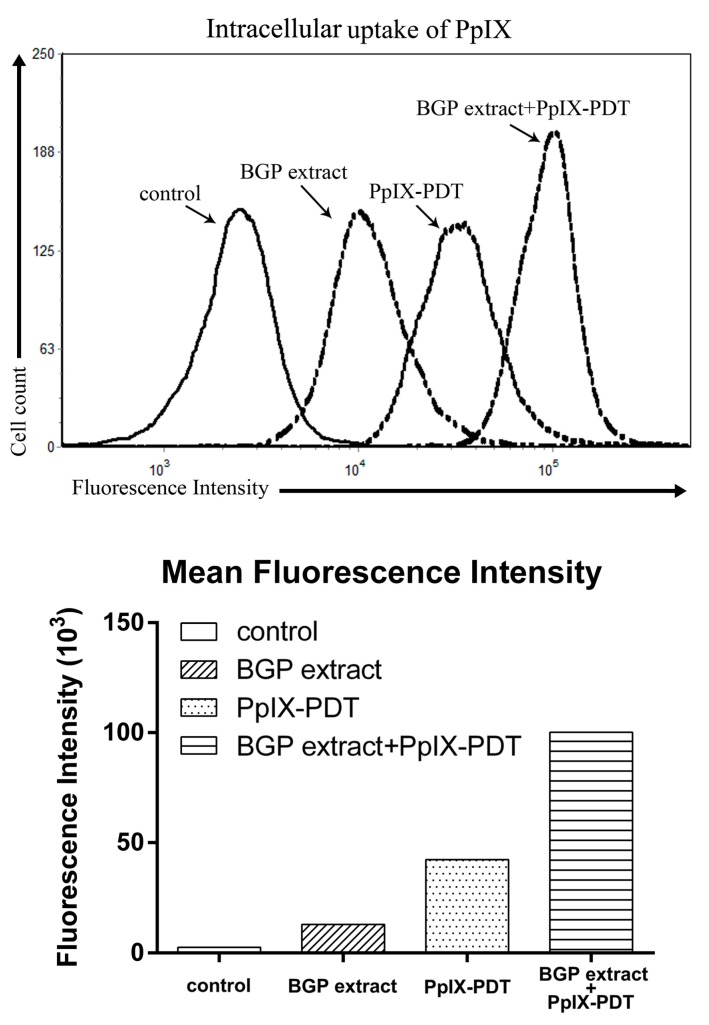
Intracellular accumulation detection of protoporphyrin IX (PpIX) by flow cytometry. Mean fluorescence intensity was measured by a BD Accuri^TM^ C6 flow cytometer.

To further investigate the synergetic mechanisms of BGP extract in PpIX-PDT, the intracellular accumulation of PpIX was examined by flow cytometry based on the fluorescence property of PpIX. Cells were treated with either PpIX or PpIX combined with BGP extract as described above. BGP extract was found to significantly enhance the intracellular accumulation of PpIX in A431 cells as a much higher PpIX specific fluorescence could be observed on the combination group compared to that of the PpIX group (Figure 7).

## 3. Discussion

Previously, propolis has been shown to augment the effect of chemotherapy on various cancers [16,29,30,31]. In this study we have investigated the synergistic effect of BGP extract on PpIX-PDT. Our findings show that BGP extract significantly synergizes with PpIX-PDT both in A431 and HeLa cells. Result in vivo is consistent with that in vitro. BGP extract increased the susceptibility of the cancer cells to apoptosis upon PDT via decreasing the anti-apoptotic protein (Bcl-xL) and increasing the pro-apoptotic proteins (Bax, NOXA and cleaved caspase-3), which indicates that BGP extract enhanced PDT-induced death through the pathways associated with mitochondrial damage.

PDT induced ROS may lead to enhanced IKKα/β phosphorylation which is an indication of NF-κB activation. It was demonstrated that NF-κB was activated upon verteporfin-mediated photosensitization in a promyelocytic cell line (HL-60 cells) [32]. An increase of NF-κB activity was demonstrated in HT-29 adenocarcinoma cells after hypericin-mediated photosensitization [33]. Korbelik has shown activation of NF-κB in photofrin-mediated PDT in a mouse SCCVII tumor model [34]. Consistent with the abovementioned studies, we found that PpIX-PDT could induce NF-κB activation in A431 cells. Since activation of NF-κB after PDT supports the survival of tumor cells by preventing apoptosis [35], pharmacological interventions in the NF-κB pathway might improve PDT outcomes.

We have explored the modulation of NF-κB pathway in PpIX-PDT upon BGP extract treatment due to its excellent antitumor activity and its unique capacity to inhibit NF-κB. As expected, we found that BGP extract attenuated the upregulation of p-IKKα/β and NF-κB induced by PpIX-PDT.

Agents targeting COX-2 were considered as adjuvants for PDT [28] as elevated levels of COX-2 were shown to decrease the efficiency of tumor treatment [36,37]. It has been reported that, combing PDT with celecoxib, a selective COX-2 inhibitor, decreased the expression of inflammatory and angiogenic factors which are frequently associated with tumor recurrence [28]. Previously, Hypericin mediated PDT in T24 and HeLa cells was shown to strongly activate the expression of COX-2 [38]. Consistent with previous findings, we found that PpIX-PDT increased the level of COX-2 whereas BGP extract clearly attenuated the induction of COX-2 by PpIX-PDT. This reduced COX-2 expression might be due to the inhibition of NF-κB by BGP extract because the promoter sequence of COX-2 contains binding sites for NF-κB, making it a downstream target of NF-κB [39,40,41]. It was reported that silencing COX-2 promotes NOXA expression [28], which is consistent with our findings that BGP extract treatment elevated NOXA expression in PpIX-PDT following COX-2 downregulation.

The tumor-selective effects of the PDT are based on the preferential uptake and retention of the photosensitizer in the tumor [42]. The intracellular uptake and accumulation of photosensitizers has been a major problem in PDT especially when treating large solid tumors [43,44]. Our results demonstrate for the first time that BGP extract increases intracellular accumulation of PpIX in cancer cells during PDT which might be another important mechanism of the synergistic effect.

Although the tumorigenic potential of cancer cells pre-treated with BGP and PDT in vitro was evaluated with a xenograft model in the present study, further in vivo studies will be necessary to demonstrate the therapeutic usefulness of BGP on PDT as our current findings dose not yet represent a proof of a true therapeutic potential.

## 4. Materials and Methods

### 4.1. Chemicals and Reagents

PpIX and 3-(4,5-dimethyl-2-thiazolyl)-2,5-diphenyl-2*H*-tetrazolium bromide (MTT) were purchased from Sigma-Aldrich (Darmstadt, Germany). TUNEL FITC Apoptosis Detection Kit was purchased from Vazyme Biotech Co., Ltd. (Nanjing, China). Annexin V-FITC/PI Apoptosis Kit was purchased from Invitrogen (Waltham, MA, USA). Antibodies against Bcl-xL, Bax, NOXA, cleaved caspase-3, p-IKKα/β, NF-κB, and anti-rabbit IgG HRP-linked antibody were purchased from Cell Signaling Technology (Danvers, MA, USA). Anti-COX-2 was purchased from Abcam (Cambridge, UK). Mouse anti-β-actin monoclonal antibody was purchased from Sigma-Aldrich. Polyclonal goat anti-mouse immunoglobulins/HRP was purchased from DAKO (Santa Clara, CA, USA).

### 4.2. Preparation of BGP Extract

The BGP sample was obtained from Royal Natural Product Co. (Markham, ON, Canada). The raw material was collected in Minas Gerais, Brazil. The raw BGP (5.0 g) were powdered and extracted with 70% ethanol twice (500, 400 mL) at room temperature for 4 h. After filtration, the ethanol extracts were combined and concentrated under reduced pressure to afford a residue (4.0 g).

### 4.3. HPLC Analysis of BGP Extract

The samples were analyzed using a LC system (Shimadzu, Kyoto, Japan) consisting of a LC-20AT binary pump, SPD-20A detector, SIL-20AC autosampler and a CTO-20AC column oven. The compounds in BGP extract were determined by HPLC-UV with a Shim-pack VP-ODS column (2.0 mm × 150 mm, 5 μm, Shimadzu). The separation was employed using 0.2% formic acid in water (A), and acetonitrile (B). A gradient elution was applied (0 min, 15% B; 5 min, 30% B; 35 min, 45% B; 45 min, 65% B; 55 min, 80% B; 60 min, 95% B.), at the flow rate of 0.3 mL/min. The injection volume was 10 μL, and the detection monitored at 280 nm. Peaks were identified, and quantified by external standard methods. The concentrated extract was dissolved in pure methanol solution at concentration level of 60 μg/mL, and then filtered through a 0.2 µm filter before HPLC analysis.

### 4.4. ESI-IT-TOF-MS Analysis of BGP Extract

MS analyses were performed using the LC-IT-TOF-MS equipped with an ESI source in negative ionization mode. The MS conditions were used as follows: detector voltage, 1.60 kV; nebulizing gas (N_2_) flow, 1.5 L/min; dry gas (N_2_) pressure, 121.0 kPa; curved desolvation line (CDL) temperature 200 °C; block temperature 200 °C; ion trap pressure, 1.6 × 10^−2^; TOF pressure, 1.3 × 10^−4^ Pa; scan range, *m/z* 100–1000 for MS^1^, 100–800 for MS^2^ and 50–500 for MS^3^; ion accumulated time was set at 30 ms for MS^1^, 50 ms for MS^2^, 70 ms for MS^3^; the collision energy was set at 50% for MS^2^ and, 30% for MS^3^. All the operations and data analysis were performed using Shimadzu LC-IT-TOF-MS solution version 3.6 and the Shimadzu Formula Predictor 1.2 was used to establish the molecular formulas.

### 4.5. Cell lines and Culture Conditions

Human epidermoid carcinoma cells, A431 and human cervical cancer cells, HeLa, purchased from KeyGen Biotech. Co., Ltd. (Nanjing, China), were cultured in Dulbecco’s modified eagle medium (DMEM) supplemented with 10% fetal bovine serum (FBS), 50 units/mL penicillin and 50 μg/mL streptomycin in 5% CO_2_ at 37 °C

### 4.6. MTT Assay

MTT assays were carried out to investigate the enhanced phototoxicity of PpIX-PDT combined with BGP extract on A431 and HeLa cells. Cells were first seeded to 96-well plates as 6000 cells/well. After 12 h, medium containing BGP extract was administrated to cells and allowed to co-incubate for another 12 h. Then a series concentration of PpIX were added accordingly and allowed to co-incubate for 5 h. Afterwards, plates were illuminated by a 450 nm laser (Banglei Photoelectronic Technology Co., Ltd., Zhongshan, China) at a power of 27 mW/cm^2^ for 30 min. The control was run in parallel. Subsequently, cells were incubated in 5% CO_2_ at 37 °C for another 48 h before MTT was added. After 6 h incubation, optical density (OD) was monitored at 570 nm by a microplate reader. Cell viability was calculated as a percentage of viable cells in drug-treated group versus untreated control by the following equation: cell viability (%) = (OD(treated) − OD(blank))/(OD(control) − OD(blank)) × 100%.

### 4.7. Evaluating the Interaction between BGP Extract and PpIX-PDT

The combination index (CI) is a quantitative measure of the degree of drug interaction in terms of additive effect (CI = 1), synergism (CI < 1), or antagonism (CI > 1) for a given endpoint of the effect measurement calculated by CalcuSyn v2.

### 4.8. TUNEL Assay

A431 cells were first seeded onto confocal petri dishes for 5000 cells per Petri dish. After 12 h, medium containing BGP extract (75 μg/mL) was administrated to cells and allowed to co-incubate for another 12 h. Then 2.7 μM PpIX was added. After 5 h, dishes were illuminated by laser at a power of 27 mW/cm^2^ for 30 min. The control group was run in parallel. The TUNEL assay was performed according to the manufacturer’s instructions. Images were captured by a confocal scanning laser microscope (IX83, Olympus, Tokyo, Japan).

### 4.9. Annexin V-FITC/PI Staining Assay

Annexin V-FITC/PI staining assay was performed to detect apoptosis. 2 × 10^5^ A431 cells were first seeded to 6-well plates. After 12 h, medium containing BGP extract (75 μg/mL) was administrated to cells and allowed to co-incubate for another 12 h. Then 2.7 μM PpIX was added. After 5 h, plates were illuminated by laser at a power of 27 mW/cm^2^ for 30 min. The control group was run in parallel. After 2 h incubation, cells were harvested and stained according to the manufacturer’s instructions. Detection was done by a BD Accuri^TM^ C6 flow cytometer (Franklin Lakes, NJ, USA).

### 4.10. Western Blotting Analysis

For western blotting analysis, cells were lysed by the addition of radio immunoprecipitation assay (RIPA) lysis buffer. Protein samples were separated by SDS-polyacrylamide gel (SDS-PAGE) under reducing conditions. Resolved protein was transferred to a methanol-activated polyvinylidene difluoride (PVDF) membrane in transfer buffer. The membrane was blocked in 5% nonfat milk for 2 h at room temperature. Then, the blocked membrane was incubated with the primary antibody at 4 °C overnight before the incubation with secondary antibody for 2 h at room temperature. Immunoreactive bands were visualized using an Immobilon Western chemiluminescent HRP substrate (Millipore, Darmstadtcity, Germany).

### 4.11. Animal Experiments

Six-weeks-old male BALB/c-nu mice, with 16–18 g, were purchased from the Model Animal Research Center of Nanjing University (Animal Certificate: SCXK Su 2015-0001) and maintained in separate cages in a specifically designed pathogen-free isolation facility with a 12 h light/dark cycle; the mice were provided rodent chow and water ad libitum. The xenograft tumor model was developed by inoculation of A431 cells. Cells were first seeded to 6-well plates. After 12 h, medium containing BGP extract was administrated to cells and allowed to co-incubate for another 12 h. Then PpIX were added and allowed to co-incubate for 5 h. Afterwards, plates were illuminated at a power of 27 mW/cm^2^ for 30 min. The control was run in parallel. 3 × 10^6^ cells after pretreatment of 2.7 μM PpIX-PDT (27 mW/cm^2^) or BGP extract (75 μg/mL) either alone or in the indicated combination were injected subcutaneously into the right flank of BALB/c-nu mice (*n* = 6). Tumor size were measured with vernier caliper once per day after one-week development, and tumor volume was calculated as V = (L × W^2^)/2, where L is the length and W is the width of the tumor. Animals were sacrificed 6 days after initial measurement and tumor weight was measured. The animal studies were carried out in accordance to conform to institutional guidelines that comply with national and international laws and policies. All animal experiments were approved by the Ethics Committee of China Pharmaceutical University.

### 4.12. Intracellular Accumulation of PpIX

Intracellular PpIX was detected by flow cytometry. A431 cells were seeded to 6-well plates. After 12 h, medium containing BGP extract (75 μg/mL) was administrated to cells and allowed to co-incubate for another 12 h. Then 2.7 μM PpIX was added. The control group ran in parallel. Cells were harvested and detected by a BD Accuri^TM^ C6 flow cytometer 5 h later.

### 4.13. Statistical Analysis

Data were expressed as mean ± SEM. Statistical significance was assessed by student test (nonparametric) followed by Welch’s correction using GraphPad Prism 6.02 (La Jolla, CA, USA).

## 5. Conclusions

Our findings provide evidence for a synergistic effect of BGP extract in PDT. BGP extract enhances the intracellular accumulation of PpIX, attenuates inflammatory factors and increases apoptosis. Based on our findings BGP should be considered as an adjuvant in future PDT approaches.
